# Accessing routinely collected health data to improve clinical trials: recent experience of access

**DOI:** 10.1186/s13063-021-05295-5

**Published:** 2021-05-10

**Authors:** Archie Macnair, Sharon B. Love, Macey L. Murray, Duncan C. Gilbert, Mahesh K. B. Parmar, Tom Denwood, James Carpenter, Matthew R. Sydes, Ruth E. Langley, Fay H. Cafferty

**Affiliations:** 1 MRC Clinical Trials Unit at UCL, UCL, London, WC1V 6LJ UK; 2 Health Data Research UK, London, UK; 3 NHS Digital, 1 Trevelyan Square, Leeds, LS1 6AE UK; 4 Medical Statistics, London School of Hygiene and Tropical Medicine, London, WC1E 7HT UK

**Keywords:** Routinely collected data, Electronic health records, Data accessibility, Clinical trials

## Abstract

**Background:**

Routinely collected electronic health records (EHRs) have the potential to enhance randomised controlled trials (RCTs) by facilitating recruitment and follow-up. Despite this, current EHR use is minimal in UK RCTs, in part due to ongoing concerns about the utility (reliability, completeness, accuracy) and accessibility of the data. The aim of this manuscript is to document the process, timelines and challenges of the application process to help improve the service both for the applicants and data holders.

**Methods:**

This is a qualitative paper providing a descriptive narrative from one UK clinical trials unit (MRC CTU at UCL) on the experience of two trial teams’ application process to access data from three large English national datasets: National Cancer Registration and Analysis Service (NCRAS), National Institute for Cardiovascular Outcomes Research (NICOR) and NHS Digital to establish themes for discussion. The underpinning reason for applying for the data was to compare EHRs with data collected through case report forms in two RCTs, Add-Aspirin (ISRCTN 74358648) and PATCH (ISRCTN 70406718).

**Results:**

The Add-Aspirin trial, which had a pre-planned embedded sub-study to assess EHR, received data from NCRAS 13 months after the first application. In the PATCH trial, the decision to request data was made whilst the trial was recruiting. The study received data after 8 months from NICOR and 15 months for NHS Digital following final application submission. This concluded in May 2020. Prior to application submission, significant time and effort was needed particularly in relation to the PATCH trial where negotiations over consent and data linkage took many years.

**Conclusions:**

Our experience demonstrates that data access can be a prolonged and complex process. This is compounded if multiple data sources are required for the same project. This needs to be factored in when planning to use EHR within RCTs and is best considered prior to conception of the trial. Data holders and researchers are endeavouring to simplify and streamline the application process so that the potential of EHR can be realised for clinical trials.

## Background

Routinely collected electronic health records (EHRs) have been identified as an important innovation in the conduct of randomised clinical trials (RCTs) [1]. EHRs could improve the efficiency and cost of trials by possibly enhancing recruitment, more complete data sets and minimal loss to follow-up [2, 3]. For example, the TASTE trial (ISRCTN16716833), using the Swedish angiography and angioplasty registry, is one of several trials demonstrating the utility of registry-held EHRs to recruit and follow up participants. This study was able to recruit 82% of eligible patients from the registry and obtained complete follow-up data in a trial of 7244 patients [4]. They also demonstrated meaningfully lower costs for managing the study with a cost per participant in the order of ~$50 compared to costs for a conventional RCT which may be in excess of $1000 per participant [4, 5].

EHRs are often collected by centralised registries and audits (national or regional) for purposes other than clinical research to gather detailed information on specific diseases, treatments or populations. However, there are concerns, depending on the source, that data collected in this way may not be of appropriate detail or quality for use in clinical trials [6]. Access to EHRs by researchers usually requires a formal application to the data holder where specific criteria must be evidenced including compliance with information governance (IG) regulations and a clear purpose and legal basis for the data access.

One potential concern for clinical trialists is that the application process will be complex and lengthy and that the data will not be obtained in a timely manner [7]. There have been reports that RCTs were unable to publish trial results due to data access [8]. One example is the EPOCH trial (ISRCTN80682973), where the research team were unable to procure mortality from Welsh data following hospital admissions. As a result, the researchers had to change their planned primary analysis to make sure their publication was not delayed significantly [9].

The aim of this article is to share and reflect upon our experience at the MRC Clinical Trials Unit at UCL (hereafter ‘MRC CTU’) in applying to three national holders of EHR datasets in the UK for data relating to two ongoing RCTs. The intention is to highlight some of the hurdles in obtaining data and discuss possible solutions. The overarching aim is to assist future applicants and help data providers, who are commonly trying to improve their processes and address these issues in a way that is mutually beneficial.

## Methods

This is a qualitative study based on recent experience of the teams at an accredited clinical trials unit (MRC CTU) in applying for and accessing routine datasets in England (for two separate trials). The data access applications are linked by one main applicant as part of their clinical methodology research and use a descriptive narrative from documented exchanges between the data holder and applicant to establish themes for discussion. These were chosen as they cover recent access to some of the main datasets likely to be used by clinical trialists with a range of common clinical outcomes. The MRC CTU sought English EHR data for the Add-Aspirin (ISRCTN 74358648) and PATCH (ISRCTN 70406718) trials.

Add-Aspirin aims to assess whether daily aspirin use after treatment for an early-stage cancer can prevent recurrence and improve survival [10]. It will recruit 11,000 participants in the UK, Republic of Ireland and India; recruitment began in October 2015 and is ongoing. The Add-Aspirin protocol includes a methodological sub-study designed to assess the feasibility of applying for and using EHRs from the National Cancer Registration and Analysis Service (NCRAS) [11] to assist in the long-term follow-up of participants after completion of trial treatment.

PATCH is a randomised trial of approximately 2500 participants with prostate cancer in the UK. It is assessing the efficacy and safety of a novel therapy transdermal oestradiol patches against standard hormone therapy [12]. Transdermal patches may have a better side-effect profile compared with standard treatment but there was a prior concern about increased cardiovascular toxicity based on trials of oral oestrogens in the 1970s. PATCH therefore had enhanced monitoring of cardiovascular outcomes, gathering all available information about each event with an additional clinical review [12]. After the trial started, a methodology sub-study was initiated to compare serious adverse cardiovascular events reported by research staff at participating sites through trial-specific data collection forms with those routinely collected from, and reported in, audits held by the National Institute for Cardiovascular Outcomes Research (NICOR) and Hospital Episodes Statistics (HES) held by NHS Digital. Concordance between the three datasets would support the premise that routinely collected data could supplement or replace long-term cardiotoxicity data in this trial and other future RCTs.

The routine data to be accessed for these two projects are held and collated by three different organisations with their own individual processes to allow data access. Although the organisations are all within the auspices of the English National Health Service, each has evolved in recent years. This, along with revisions to the legal framework for IG, means that the process of data access has also evolved.

### National Cancer Registration and Analysis Service (NCRAS)

In 2016, NCRAS was formed from the merger of the National Cancer Intelligence Network (NCIN) and National Disease Registration (NDR) within Public Health England [13]. In England, NCRAS manages the collection of data relating to cancer. The aim is to monitor cancer incidence, improve care and clinical outcomes, aid research and support genetic counselling [11]. NCRAS hold several different datasets covering cancer registration and cancer treatments (systemic therapy and radiotherapy). They can also link these datasets to others held by NHS Digital or the Office for National Statistics (ONS), such as mortality data and HES, via NHS number or other personal identifiers.

**Fig. 1:**
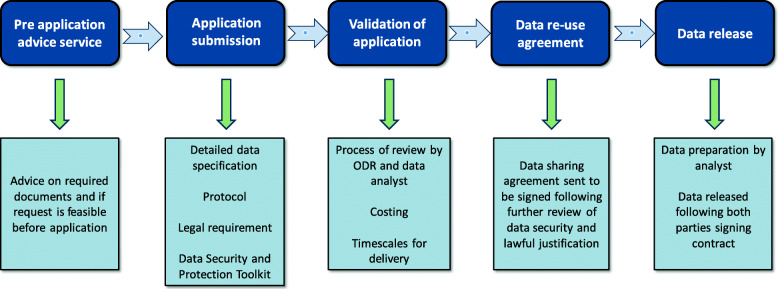
Flow diagram of data access via the Office for Data Release (ODR) for National Cancer Registration and Analysis Service (NCRAS) data, adapted from Public Health England (PHE) [14]

To gain access to this data for research, an application must be submitted to the Office for Data Release (ODR) [14]. The ODR application process is outlined in Fig. 1 [14].

### NHS Digital

**Fig. 2:**
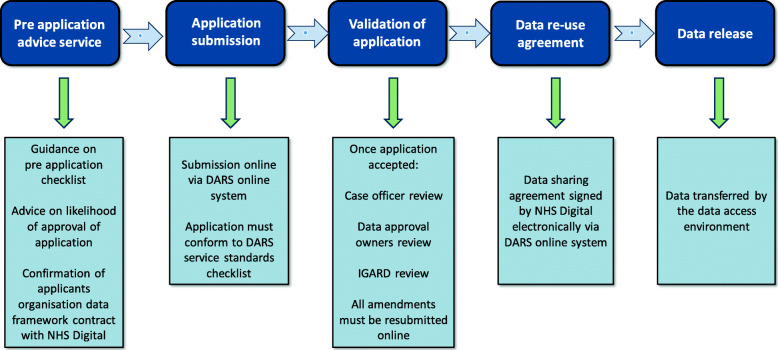
Flow diagram of the Data Access Request Service (DARS) for NHS Digital data, adapted from NHS Digital [17]. IGARD, Independent Group Advising on the Release of Data

NHS Digital has been the custodian of HES since 2016. Prior to this, it operated under the Health and Social Care Information Centre (HSC-IC) from 2005 [15]. NHS Digital collects, processes and provides access to many EHR datasets and is continually seeking to supplement this data with other datasets from various care settings. HES is primarily a resource for reimbursement of hospital activity and holds patient-level information on more than 500 variables ranging from diagnosis, procedures, admission dates, demographics of the patients and healthcare provider [16]. NHS Digital has a large number of organisations requesting access to their data with most coming from local authorities and Clinical Commissioning Groups [8]; access is provided by application to the Data Access Request Service (DARS) [17]. The Independent Group Advising on the Release of Data (IGARD) gives an independent final review that aims to improve transparency, accountability, quality and consistency of the application process. IGARD currently meets weekly to make sure that applications are reviewed in a timely fashion. The application process continues to change with attempts to improve its service; the current process is outlined in Fig. 2 [17].

### National Institute for Cardiovascular Outcomes Research (NICOR)

**Fig. 3:**
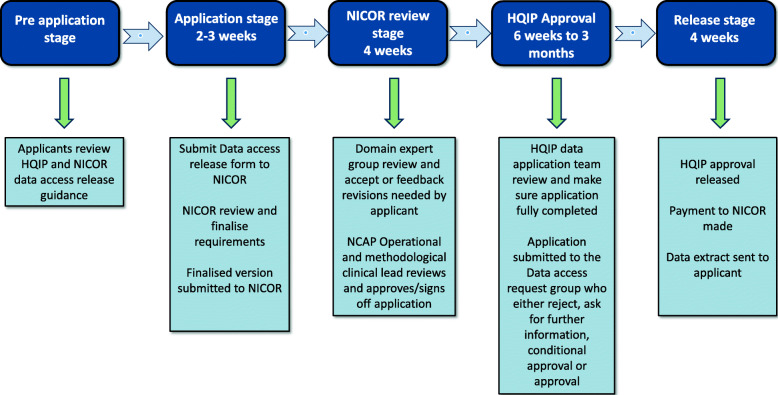
Data access request for access to National Institute for Cardiovascular Outcomes Research (NICOR) data adapted from [18]. HQIP, Health Quality Improvement Partnership; NCAP, National Cardiac Audit Programme

NICOR collects routine EHR data and produces analyses to enable hospitals and healthcare improvement bodies to monitor and improve the care and outcomes of patients with cardiovascular disease. It manages six national clinical audits and a number of new health technology registries [18]. NICOR is regulated and contracted by the Health Quality Improvement Partnership (HQIP). NICOR was originally hosted by UCL but moved to Barts Health NHS Trust in 2017. The two audits that were identified as potentially relevant to the PATCH trial were the National Heart Failure Audit (NHFA) and the Myocardial Ischaemia National Audit Project (MINAP). The application process to obtain data from NICOR is shown in Fig. 3 [18]. Historically, far fewer researchers have used this source compared to NHS Digital and NCRAS [8].

## Findings

### Add-Aspirin

**Fig. 4:**
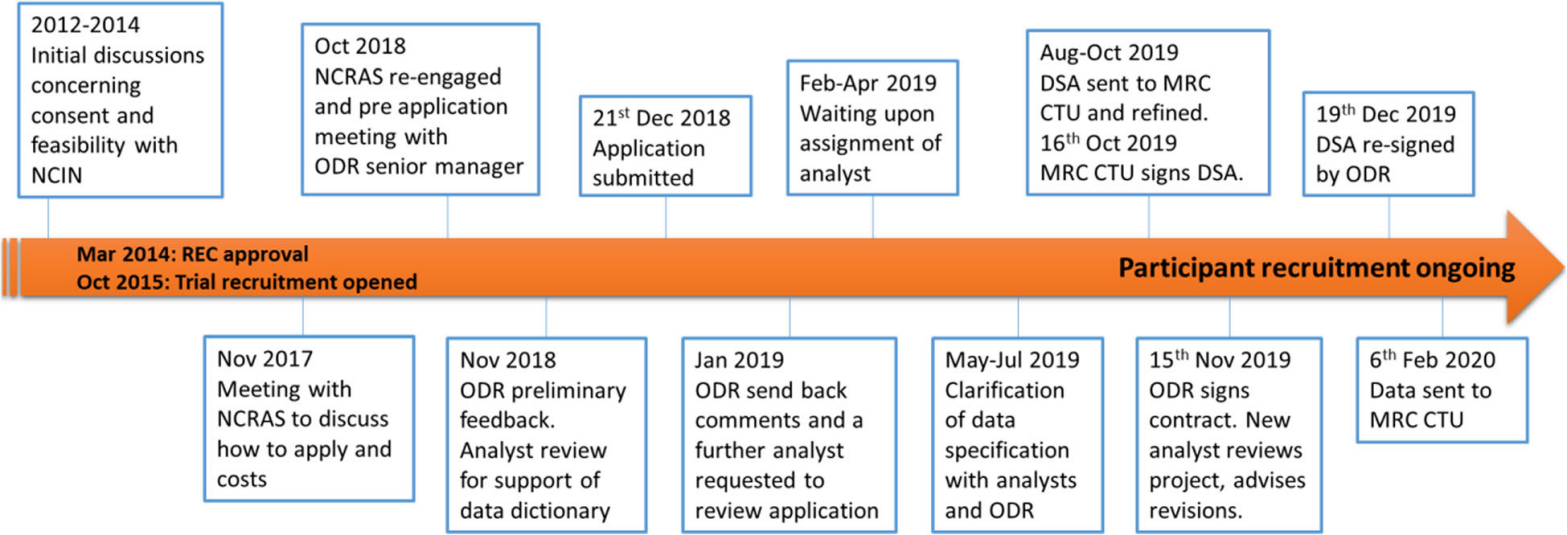
Flow diagram of the Add-Aspirin National Cancer Registration and Analysis Service (NCRAS) application. (Please note that timeline is not proportional) REC approval for Add-Aspirin March 2014. Recruitment opened in October 2015 and is ongoing. CTU, clinical trials unit; DSA, data sharing agreement; NCIN, National Cancer Intelligence Network; ODR, Office for Data Release; REC, Research Ethics Committee

The Add-Aspirin trial was conceived with the recognition that participants will require follow-up for at least 10 years [10]. This length of follow-up is required to assess the overall risk: benefit of regular aspirin use on the trial participants’ health. From the design stage of the trial, like for many trials [19], there was an intention to access data using routinely collected EHRs. When the trial was initially conceived in 2012, the Add-Aspirin trial team met with individuals from NCIN, the predecessor of NCRAS, to assess the feasibility of accessing data and also to ensure that an appropriate budget for this activity was incorporated into funding applications (Fig. 4). The protocol, patient information sheets and consent forms were designed to reflect the potential use of routinely collected healthcare data.

In 2017, after 2 years of recruitment and follow-up, there was a conversation with ODR to confirm the cost and current application process. In 2018, there was sufficient data to initiate the pre-defined methodology sub-study. A pre-application meeting with an ODR senior manager established the documentation that was needed going forward.

Following the implementation of the General Data Protection Regulation (GDPR) in the UK (2018), transparency of how exactly participant data would be used became a legal requirement. The previously agreed consent forms and patient information sheets did not meet the 2018 requirements of GDPR. The solution was for a privacy notice to be drafted and made publicly accessible on the trial’s website. The trial’s IG documentation also needed updating to ensure information security assurances (via the Data Security and Protection Toolkit) were in place within UCL.

Following submission of the data application (December 2018), ODR sent back revisions (January 2019) and confirmed the transparency statement (February 2019). For the application to proceed, an analyst needed to be allocated to check the defined data requirements. In April 2019, NCRAS unfortunately unassigned the analyst allocated to Add-Aspirin onto work on a project considered more critical. There was a meeting in May 2019, once further analytical support had been deployed, to discuss the data field requests. The new analysts suggested that a number of data fields should be expanded to give the best chance of capturing cancer recurrence as this is not, at present, collected sufficiently well within any single EHR dataset. They acknowledged at that time that algorithms were needed to identify data patterns indicative of tumour recurrence. ODR wanted to ensure that no unnecessary data from HES was provided for each participant. The MRC CTU therefore provided surgical/procedure codes (using Office of Population Censuses and Surveys (OPCS) definitions) and diagnosis codes (ICD-10 codes) to NCRAS to focus and limit the data extraction. In June 2019, it was agreed with ODR and NCRAS that, as this was a methodological project reviewing ways to gather trial outcomes in registry data, all HES data for these patients could be given to the MRC CTU.

The application then underwent an ODR internal moderation review, and a month later, a data sharing agreement (DSA) was sent from ODR to MRC CTU. Between August and October 2019, there were ongoing discussions between the MRC CTU contracts department and the ODR. The final DSA was signed on behalf of MRC CTU on 16 October 2019 and fully executed by ODR on 15 November 2019. A further new analyst was then assigned to the project who re-reviewed the data request. This new analyst advised an update to the data censor dates, since more up-to-date data was now available from NCRAS. The updated data request was sent back to ODR for re-signing. The DSA was re-signed and the MRC CTU checked the current consent status of patients before sending participants identifiable data to NCRAS on 23 December 2019. The one-off data extracts were successfully received at the MRC CTU on 06 February 2020. This 6-week interval before data receipt was due to NCRAS rewriting their standard filters to provide C44 (non-melanoma skin cancer) — a code that is not usually supplied but needed for this trial. In total, this application, excluding the planning and preparatory work, took approximately 13 months from submission of the application to receiving the data.

### PATCH

The PATCH trial opened to recruitment in 2006 as a phase II feasibility trial, developing into a phase III RCT in 2013. The trial was not initiated with the use of EHR in mind but there was a statement included in the consent form to potentially allow information to be sought from the national registries in the future:

I agree that my details including my full name can be given to the MRC such that long-term follow-up information from the NHS Information Centre and the NHS Central Register or any applicable NHS information system.

**Fig. 5:**
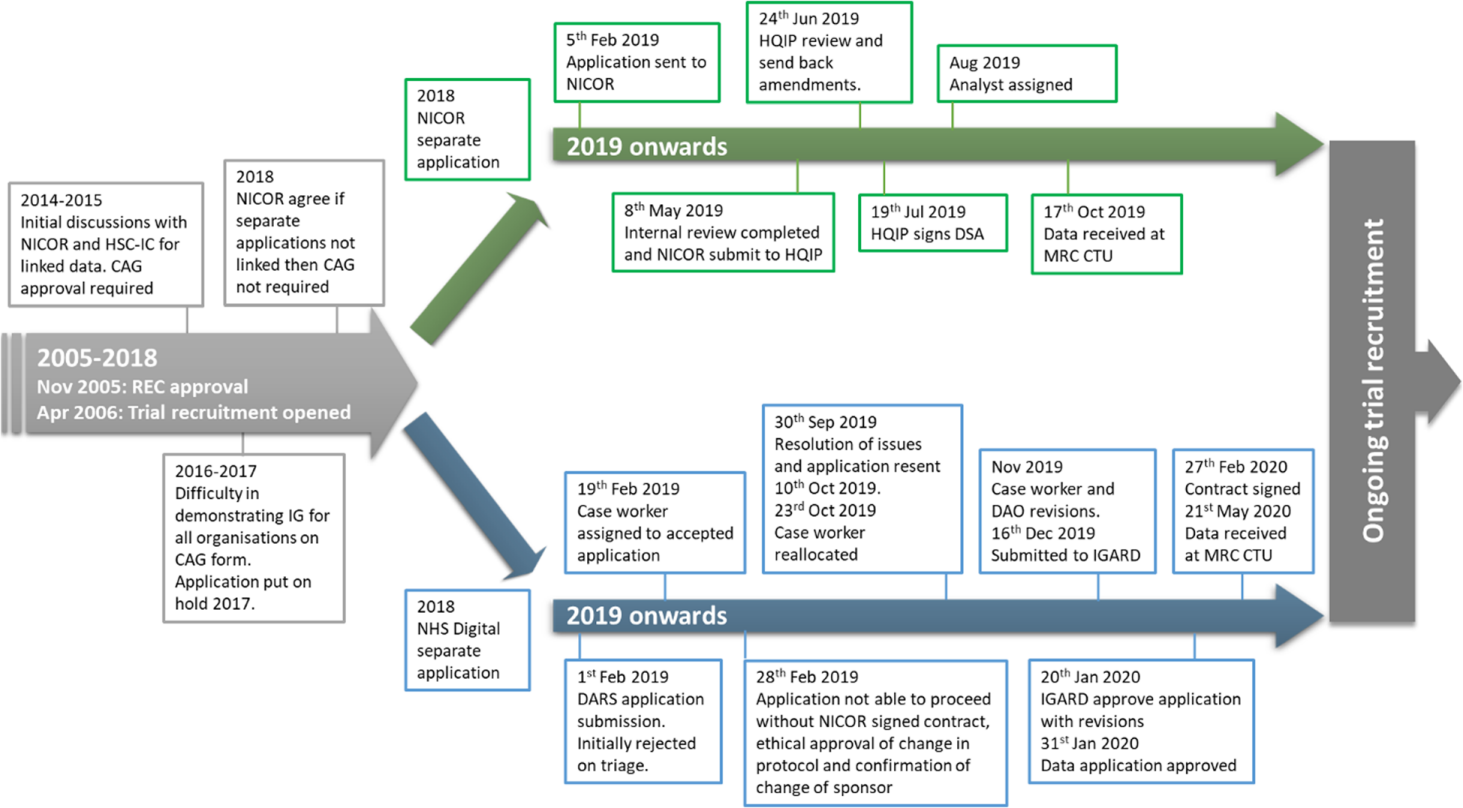
Flow diagram of the PATCH joint application to NHS Digital and National Institute for Cardiovascular Outcomes Research (NICOR) and subsequently handled as separate applications in 2018. (Please note that timeline is not proportional) REC approval for PATCH November 2005. Recruitment opened in April 2006 and is ongoing. CAG, Confidentiality Advisory Group; DAO, data approvals officer; DARS, Data Access Request Service; HQIP, Health Quality Improvement Partnership; HSC-IC, Health and Social Care Information Centre; IGARD, Independent Group Advising on the Release of Data; REC, Research Ethics Committee

With the assumption of valid consent for the use of EHR data, a methodological sub-study was devised to triangulate cardiovascular event data between HES, NICOR and trial data. There was an initial scoping of the project in 2014 with NICOR and HSC-IC advising data linkage before comparison at the MRC CTU (Fig. 5). During the initial conversations with NICOR and HSC-IC, the organisations stated that the consent statement was insufficient to acquire linked data from these two sources without first gaining approval from the Confidentiality Advisory Group (CAG). In 2016, the process to submit a CAG application was started. Several months of delays followed due to difficulty in acquiring the appropriate IG documentation for PATCH. CAG require detailed IG documentation for both the trial but also in this case from NICOR and NHS Digital (formerly HSC-IC until 2016). There were difficulties in identifying the appropriate person for this information within NHS Digital, taking most of 2016 to achieve (note: at this time, case officers were not assigned until after the application was formally submitted). During 2016, an alternative method of data access was explored via NCRAS, but as no cancer data was being sought, this option was deemed unviable. Consequently, in 2017, the project was put on hold.

In October 2018, the MRC CTU re-engaged with NICOR (which had moved to Barts Health NHS Trust following a European Union tender process) and NHS Digital. There were additional complexities for obtaining CAG approval as the PATCH trial at the time was in the process of changing sponsor and therefore the CAG application could not be approved.

As the explicit wording on the consent form was the main issue preventing access to the data, the MRC CTU asked the MRC Regulatory Support Centre for further guidance. They felt that the consent wording was sufficient. NICOR subsequently agreed that, if their data was not sent to NHS Digital for linkage, then CAG approval was not necessary. Therefore a further application was submitted and sent to NICOR for review (Fig. 3). NICOR’s review was completed in May 2019. The application was then submitted to HQIP by NICOR. The application was reviewed in June and amendments were returned to MRC CTU. HQIP issued a signed DSA on 19 July 2019, and a NICOR analyst was assigned. The analyst continued discussions with the MRC CTU on data extraction, and a one-off data extract was received at the MRC CTU on 17 October 2019.

As with NICOR, NHS Digital was re-engaged in October 2018, and it took several weeks to allow access to the DARS online system due to technical difficulties with the DARS system (Fig. 5). A new DARS application was submitted in February 2019, but this was initially rejected due to issues around consent and sponsorship and not meeting the DARS checklist criteria. After a phone call to DARS and changes to the application by the MRC CTU, it was accepted and a case officer allocated. The case officer reviewed and made extensive comments with required changes. A privacy notice was created for the project and circulated to participants once it was ethically approved. NHS Digital then advised that the application could not proceed until the NICOR DSA was signed, sponsorship clarified and the new protocol for the sub-study had been ethically approved.

Sponsorship was not resolved until September 2019, and at that point, the MRC CTU re-engaged with NHS Digital. On receipt of the revised application, NHS Digital returned it to the DARS triage service and a new case officer was allocated. Over the next few months, the case officer made amendments to the application and sent it internally to the data approvals officer (DAO). The DAO asked for further changes to the application to clarify certain points and was submitted to IGARD in December 2019 for final review. IGARD approved the application in January subject to one last data specification amendment. The DSA was signed on behalf of the MRC CTU in February 2020, and the MRC CTU uploaded identifiable data to NHS Digital in March. The NHS Digital production team made data available in May and data was received at the MRC CTU on 21 May 2020. When all efforts are taken into consideration, it has taken several years to obtain data from both of these providers. However, from the most recent effort, data was received approximately 8 and 15 months after submission of formal applications to NICOR and NHS Digital respectively.

## Discussion

This article describes the MRC CTU’s experience of attempting to access EHR data from three English national data holders (NCRAS, NICOR and NHS Digital) for two large trials with a view to identifying shareable lessons. These data access applications were chosen as they were both for methodological studies embedded within RCTs looking at the appropriateness of EHR data to be used in trial follow-up with the important juxtaposition of where data access is planned versus being a later addition. The aim was to improve the knowledge and experience of gaining access to these datasets and to assess the accuracy of nationally held EHR data compared to data manually collected as part of conventional trial-specific follow-up. Our experience was challenging and took many person hours over 8 to 15 months from formally submitting an application to receiving the data.

There are limitations to this paper as this is specific to English national data holders and other countries may not have the same application issues or comparable registry data quality. This is also an experience paper from one clinical trials unit, and the difficulties we had in acquiring the data may potentially be unique. The nature of the trials, the infrastructure within this specific trials unit, the introducing of significant data protection legislation (GDPR; May 2018) during the period that provide new requirements, and the relative infrequency of our applications could be factors in the delays encountered. The process of applying for data for the PATCH trial started more than 5 years ago but the most recent iteration of applications for data started in October 2018. However, this is not a story in isolation and there have been other publications demonstrating similar problems [7, 9, 20, 21]. At present, the application process for each of these datasets is too complicated and discourages researchers from using this invaluable data. A recent survey of the cancer research community, conducted by the National Cancer Research Institute, found that less than half were successful in accessing data from the national datasets and, when asked what would help most, the majority answered ‘support through data access process’ and ‘improving timelines for the application approval’ [22]. The difficulty of accessing this data may be why so few clinical trials have used national datasets to enrich or replace data collected via conventional case report forms [8].

From a clinical trialist perspective, several lessons have been learnt about the process of applying for and obtaining EHR data. Firstly, it is extremely challenging to acquire data for an actively recruiting trial that had not planned this acquisition in advance. The main issue for the PATCH trial application was the wording in the trial protocol, consent forms and patient information sheets were not initially designed for the sub-study when the application process was started. Although the wording followed current recommendations when first written, information governance procedures and regulations evolved. In contrast, the Add-Aspirin trial had a good foundation due to prior preparation work before the application process began which meant fewer amendments were needed due to new data laws. Clinical trials units need to work closely with registries and data holders to establish the most efficient methods to obtain and access EHR data; this could include clear guidance on the optimal timing of data requests (such as at trial initiation) and accessible, transparent cost structures to allow trialists to obtain sufficient funding for repeated data access through the lifetime of a trial.

Secondly, all clinical trials units need appropriate infrastructure to have the high level of data security needed for storing EHR data, and evidenced through a completed and endorsed Data Security and Protection Toolkit. An example includes the formation of ‘Trusted Research Environments’ which allow a cyber-secure virtual location where identifiable data cannot be removed and only verified researchers can access depending on IG training and specified parameters. Such infrastructure is complicated and costly taking considerable time to set up and to manage going forward. Once the required infrastructure is established, then the data security and IG controls should be valid for any national dataset. The connectivity of these datasets is also an issue, with separate applications having to be completed to several organisations/countries within the UK which takes a considerable amount of time and money. One solution would be a ‘passport’ system for data access to allow an institution that has demonstrated appropriate data security and IG controls to fast track the process. Another solution would be to link more datasets and allow only one application for both. There are new initiatives ongoing with examples of collaboration such as VICORI which links between NICOR and NCRAS data [23].

Lastly, the applicant also needs experience in how to answer the questions in the forms to stand up to the scrutiny of the data controllers’ checks. These assessments are appropriate but, without prior knowledge, applications are often rejected due to wording rather than due to the nature of their request. This could make it difficult for clinical trials units that only apply occasionally since key knowledge may be lost inducing repetitive errors again, or the team is unaware of how the process has changed. This lack of experience can only be helped by resources provided by the dataset organisations and more guidance through the application process by experienced case officers within those organisations.

NHS Digital and NCRAS are continuing to improve their accessibility through guidelines for the application process, seminars and videos. NHS Digital has established a clinical trials service in collaboration with Health Data Research UK, the University of Oxford, IBM and Microsoft [24]. This ‘NHS DigiTrials’ is in its infancy and is initially concentrating on helping new trials with the identification of potential participants and follow-up of participants during and post-trial. As part of this, it is directing its attention to helping with data access from EHR for clinical trials by increasing the speed of access and a wider range of data types available. NICOR are also striving to streamline their application process internally and with HQIP to avoid unnecessary delays for appropriate research applications. During the COVID pandemic, there has also been data sharing and routine linkage for the first time between NICOR and NHS Digital that has been used in a number of publications [25].

For routinely collected EHR to be a viable option of providing data for clinical trials, data access must take no longer than a few months; otherwise, delays cause difficulty with funding and the timeliness for reporting key outcomes. Also, the records within the databases need to be up-to-date. Some may have a reporting lag of up to a year and that limits their utility. Also, better coordination and linkage between the datasets held by separate data controllers would reduce the burden on the applicants. Health Data Research UK (HDR UK) is working with key stakeholders to improve data ‘inclusivity and transparency’ to push the agenda of utilisation of data for science with relevant organisations but also with the public as well. This also includes improving navigation across datasets from different data controllers, via the Health Data Research Innovation Gateway, and bringing together different data controllers under the UK Health Data Alliance [26]. This is to be consistent with their bold statement of ‘Our Data, Our Society, Our Health’ [20]. This will hopefully allow the right data to be given to the right people in an efficient but transparent way and provide reassurance to the general public. The accessibility is the first challenge in the use of this data but there is still concern about how appropriate the data is, given that it is not designed for clinical trials. Evaluation of the reliability, completeness and accuracy of data is needed. The analysis of the EHR data of the two methodology projects described above is ongoing and will be the subject of separate publications which will further inform the discussion around the utility of EHR in trials.

## Conclusion

EHR contains a wealth of information about individual patient’s health outcomes, which can be useful for clinical trials. Our experience demonstrates that data access can be a prolonged and complex process. This is compounded by the fact that multiple data sources, sometimes from different data holders, will often be required for the same project. Improving data access would be the first step to realise the potential of these datasets. Based on our experience successfully accessing datasets from NHS Digital, NCRAS and NICOR, we have identified pre-planned acquisition of data prior to trial set up is important for researchers considering the use of EHR data for their clinical trials to establish appropriate consent, legal purpose and infrastructure to comply with data security and law. Data holders and researchers are endeavouring to simplify and streamline the application process so that the potential of EHR can be realised for clinical trials.

## Data Availability

Data sharing is not applicable to this article as no datasets were generated or analysed during the current study.

## Abbreviations

CAG: Confidentiality Advisory Group of the Health Research Authority (HRA)
CTU: Clinical trial unit
DAO: Data approvals officer
DARS: Data Access Request Service
DSA: Data sharing agreement
EHR: Electronic health record
GDPR: General Data Protection Regulation (EU) 2016/679; implemented 25 May 2018
HDR UK: Health Data Research UK
HES: Hospital Episodes Statistics
HQIP: Health Quality Improvement Partnership
HSC-IC: Health and Social Care Information Centre
IG: Information governance
IGARD: Independent Group Advising on the Release of Data
MINAP: Myocardial Ischaemia National Audit Project
MRC CTU: Medical Research Council Clinical Trials Unit at UCL
NCAP: National Cardiac Audit Programme
NCIN: National Cancer Intelligence Network
NCRAS: National Cancer Registration and Analysis Service
NDR: National Disease Registration
NHFA: National Heart Failure Audit
NICOR: National Institute for Cardiovascular Outcomes Research
ODR: Public Health England (PHE) Office for Data Release
ONS: Office for National Statistics
OPCS: Office of Population Censuses and Surveys
Privacy notice: A statement made to a data subject that describes how the organisation collects, uses, retains and discloses personal information; also known as a transparency notice
REC: Research Ethics Committee
